# How children with juvenile idiopathic arthritis view participation and communication in healthcare encounters: a qualitative study

**DOI:** 10.1186/s12969-021-00642-x

**Published:** 2021-11-02

**Authors:** Veronica Lundberg, Catharina Eriksson, Torbjörn Lind, Imelda Coyne, Anncristine Fjellman-Wiklund

**Affiliations:** 1 Department of Community Medicine and Rehabilitation, Physiotherapy, Umeå University, SE 901 87 Umeå, Sweden; 2 Department of Clinical Sciences, Pediatrics, Umeå University, SE 901 85 Umeå, Sweden; 3 Department of Public Health and Clinical Medicine/Rheumatology, SE 901 87 Umeå, Sweden; 4 School of Nursing & Midwifery, Trinity College Dublin, Dublin, Ireland

**Keywords:** Adolescent, Child, Juvenile idiopathic arthritis, Parent, Participation, Qualitative, Young adults

## Abstract

**Background:**

Children report that they do not participate in their healthcare as much as they want, despite having the lawful right to form their own views and the right to express those views freely in all matters affecting them. Children and parents appeared to be more satisfied when healthcare professionals (HCP) use a participatory style in healthcare encounters.

**Aim:**

To explore how children, adolescents and young adults with Juvenile Idiopathic Arthritis (JIA) and parents of children with JIA view their participation and communication in healthcare encounters with healthcare professionals.

**Methods:**

Using a qualitative study design, participatory workshops were held separately for children and young adults with JIA and parents of children with JIA. The workshop data were analysed with Graneheim and Lundman’s Qualitative Content Analysis resulting in one main theme and two subthemes.

**Results:**

The theme “Feeling alienated or familiar with healthcare encounters” illuminates how children felt alienated at healthcare encounters if they found the encounters emotionally distressing. Children could withhold information regarding their health and function from both HCPs and their family and friends. The subtheme “Distancing oneself from healthcare” describe why children felt reluctant to engage in the healthcare encounters and experienced difficulty expressing how they really felt. The subtheme “Being a normal event in life” describe how children felt more comfortable over time engaging with HCPs when they knew what would happen, and felt that HCPs gave them the necessary support they needed to participate.

**Conclusions:**

Children’s participation in healthcare encounters varied depending if children felt alienated or familiar to the healthcare situations. Children distance themselves and are reluctant to engage in healthcare encounters if they find them emotionally distressing and feel disregarded. Over time, children can become more familiar and at ease with healthcare situations when they feel safe and experience personal and positive encounters. When the children are prepared for the encounter, provided with the space and support they want and receive tailored help they are more enabled to participate.

## Key messages

1. Children’s participation in healthcare encounters varies depending on if children feel alienated or familiar to the healthcare situation.
2. Children may distance themselves from healthcare encounters if they find the encounters emotionally distressing, feel disregarded and feel labelled.
3. Children need a sensitive welcoming encounter and to trust the good intentions of the healthcare professional so they can freely express their concerns and needs.
4. Healthcare professionals need to be aware that children may withhold information because they feel unsupported, want to protect their family and friends, or are afraid of the consequences a disclosure can have.

## Introduction

JIA is a chronic inflammatory joint condition of unknown origin with onset before the age of 16 years and persisting more than 6 weeks. The condition may result in functional disability, pain, lifelong medication use and school absences, all of which negatively affect children’s health-related quality of life (HRQOL) [1]. The children strive for normality and can have difficulty taking their medicines since the medications impact the children both socially and personally [2, 3]. Parents of children with JIA can perceive their children as being more vulnerable compared to parents of healthy children [4].

This article relates to children’s rights to understand the communications and participate as much as they want to in healthcare encounters. Every child who is capable of forming their own views shall have the right to express those views freely in all matters affecting them, according to Article 12 in the United Nations Convention of the Rights of the Child (UNCRC) [5]. The Convention was adopted by UN General Assembly in 1989, and became a law in Sweden on 1st of January 2020 which means that children’s rights are legally strengthened [6]. Despite a 30 year-period since ratification, children continue to report that they cannot participate in their healthcare as much as they prefer [7–9]. Participation can be defined as actively taking part in healthcare encounters and communication with healthcare professionals (HCP) [10]. Implementing the United Nations Convention seems to be challenging for HCPs and the organisations they work in [11]. It is crucial to understand that Article 12 is a legally binding obligation, not something gifted by adults [11], thus HCPs need to develop strategies to promote children’s participation and include them in their own healthcare [12–14].

Children in a healthcare setting can be perceived as in need of protection when they are unwell, in an unfamiliar environment and lack knowledge of medical matters [15]. Adults’ judgements of what is best for the child is not necessarily in accordance with the child’s own interests [15]. Acting in a child’s best interest means enabling the child to make the child’s views be heard alongside the views of parents and HCP [15]. Chronically ill adolescents need to be encouraged to communicate and become more independent at healthcare encounters, also in preparation for transition to adult care services [8]. Children and parents are more satisfied when HCP are using a participatory style and include them in discussions about the child’s treatment regimen [9].When children are invited to participate in conversations with HCP, their self-confidence is enhanced and they can feel strengthened, supported and respected [12]. It is important to increase the knowledge about children’s experiences and preferences regarding participation and communication in healthcare encounters.

Therefore, the aim of this study was to explore how children, adolescents and young adults with JIA and parents of children with JIA view participation and communication of children and adolescents, in healthcare encounters.

## Methods

### Study design

**Fig. 1:**
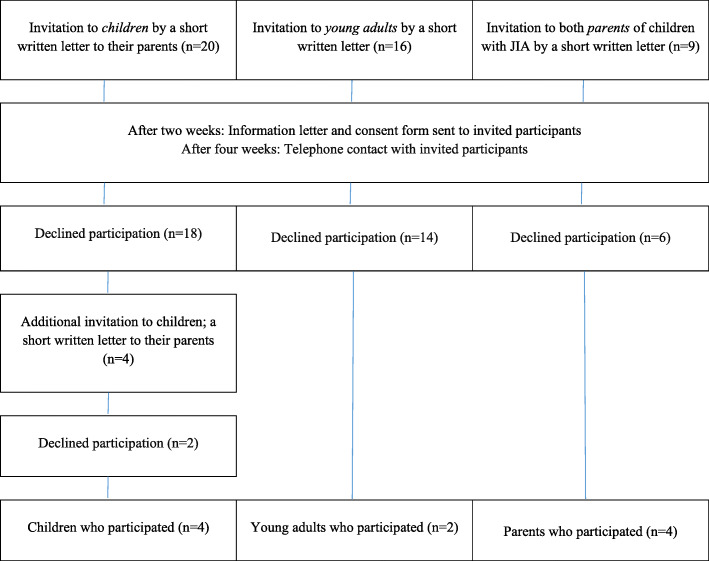
Flow chart of recruitment process of participants to separate workshops

This study is an exploratory qualitative study based on workshops with children, adolescents and young adults with JIA, and parents of children with JIA. Participants were chosen through purposive sampling [16] and a total of 10 participants accepted (see recruitment process outlined in Fig. 1).

Participants were recruited through a child and adolescent centre at a Swedish tertiary hospital. Inclusion criteria for children were; aged 10-17 years, both girls and boys, who lived within 50 km from the hospital. The children and young adults did not all have the same JIA sub-diagnosis and they had been diagnosed with JIA for more than one year and up to 23 years.

For workshops with young adults with JIA, sixteen persons were recruited via former participation in a cross-sectional study between 2009 and 2011 [17, 18]. Parents of nine children with JIA were asked to participate. Four parents to children diagnosed with JIA for over a year accepted and participated in the workshops, of which both parent of one child participated.

### Data collection

Data were obtained from children, young adults and parents in separate workshops and were conducted at a Unit for Play therapy, at a Swedish tertiary hospital and led by the lead author. In the child and parents’ workshops, RJ or AFW accompanied VL. Each separate workshop started with VL explaining the aim of the study, background, participants’ rights and the format and purpose of the workshop.

Participating young adults were asked to recall experiences from healthcare encounters as child. The two workshops with parents of children with JIA, had the same format as with the workshops for young adults (Table 1). The workshops lasted for 60-70 min for children and 90 min for young adults and parents. All the workshops were audio-recorded and transcribed verbatim by a professional transcriber.

**Table 1:** Format and purpose of workshops with children and young adults with JIA and parents

Time of workshop	Workshop	Format	Purpose
*Autumn 2017*	*Child workshop 1, with two participants*	A “get to know each other exercise”, followed by a film about a girl with JIA. Questions based on the film.	Explore children’s views on how HCP can facilitate for children to communicate how they feel, and to not be nervous before healthcare encounters
	*Child workshop 2, with two participants*	Home assignment with drawings and/or text	Explore how children feel before, during and after healthcare encounters. How they would like to feel, and what was needed to feel that way
*Spring 2018*	*Child workshop 3, with three participants*	Mind-map on “A dream child and adolescent outpatient setting”	Explore children’s wishes about healthcare environment, HCPs and the healthcare encounters
	*Child workshop 4, with two participants*	Written scenario about a child at a healthcare encounter. Children filled in the “gaps” in the story	Explore children’s experiences, wishes and needs at healthcare encounters
	*Parent workshop 1, with three participants*	Mindmap; a good healthcare encounter for their child	Explore parents views of their and their children’s preferences at healthcare encounters
	*Parent workshop 2, with four participants*	Mindmap; “the good meeting”	Explore parents views of which support and needs their children require to experience “a good encounter”
*Autumn 2018*	*Young adult workshop 1*, *with one participant*	Mindmap; a good healthcare encounter as a child	Explore recall of positive experiences and participation at healthcare encounters as a child
	*Young adult workshop 2*, *with two participants*	Mindmap; “the good meeting”	Explore experiences of which support and needs could facilitate participation at encounters as a child
*Spring 2019*	*Child workshop 5, with one participant*	Presenting of preliminary results from previous workshops	Feedback on preliminary results
	*Parent and Young adult workshop 3, with one parent and one young adult*	Presenting of preliminary results from previous workshops	Feedback on preliminary results

### Data analysis

Transcripts of the workshops were analysed with Qualitative Content Analysis (QCA) according to Graneheim and Lundman [19, 20]. QCA is useful for analysing experiences, reflections and attitudes of people or groups [21, 22]. The workshops were analysed using an inductive approach. QCA focuses to a great extent, on the subject and context, and emphasises differences between and similarities within codes and categories. The manifest content, what the core text says, is often presented as categories, while the latent content, that is what the text is talking about and can be seen as ”the red thread” through the text is presented as themes [19].

The data analysis started with VL listening to each recorded workshop and reading through the transcripts looking for inaccuracies. Transcripts were further read repeatedly as a whole and in parts. All information related to the aim was given codes. Codes with similar content were clustered and formed into sub-subthemes, subthemes and a theme on an abstract and interpretative level. The analysis was followed by continuous mutual discussions of the results with AFW to negotiate outcome. In the last step the results were discussed with the remaining three authors (CE, TL, IC). VL and AFW are physiotherapists, CE and TL are physicians, IC is a children’s nurse. VL, AFW and IC have expertise in qualitative studies. VL, CE, TL and IC have specialised in paediatrics and JIA.

## Results

The analysis resulted in the theme “Feeling alienated or familiar with healthcare encounters” with two subthemes: “Distance oneself from healthcare” and “Being at ease with healthcare encounters” (Table 2). The subheadings represent the theme and subthemes. Quotations from participants are presented in italics, or within text as short quotes. Child quotations include both children’s, adolescents’ and young adult’s quotations.

**Table 2:** The themes presenting children, young adults and parents’ experiences regarding child participation in healthcare

Examples of codes	Sub-subtheme	Subtheme	Theme
Nervous about bad news	Stressful healthcare encounters	Distancing oneself from healthcare encounters	Feeling alienated or familiar with healthcare encounters
Do not understand	Feeling disregarded		
Don’t want to worry parents	Withholding information		
Know what to expect	Being a normal event in life	Being at ease with healthcare encounters	
Come by yourself: dared to ask	Enabled by physical and mental space		

### The theme – feeling alienated or familiar with healthcare encounters

This theme illuminates how children felt alienated at healthcare encounters if they felt disregarded and found the encounters emotionally distressing. Children could withhold information regarding their health and function from both HCPs and their family and friends. In order to feel at ease with healthcare encounters, children required extra support from the HCPs and their parents, especially when they were young, and during the first period after diagnosis. The children also needed to feel understood and respected, safe with the HCPs, and that they received the help they needed. If their needs were met, the children could feel familiar with the healthcare encounters and *‘grow in to it’* (Table 2).

### Distancing oneself from the healthcare encounters

This subtheme consists of the three sub-subthemes: “Stressful healthcare encounters”, “Feeling disregarded” and “Withholding information”. These describe why children felt reluctant to participate in healthcare encounters and to express how they really felt.

Children distanced themselves from healthcare when they found the encounters emotionally distressing. They were unsure of what would happen, felt they had no control over the situation and worried they would hear *bad news, like things have gotten worse maybe. New medications”*, and would need to provide blood samples. Having to undress in front of several staff, students and their parents were perceived as violating privacy, and the child needed to *“turn off and try not to be bothered, but it was still uncomfortable”*. Likewise, having to weigh in at each encounter affected adolescents negatively. The children thought that the healthcare encounters were time-consuming and boring and they *“just wanted to get it over with”* or be elsewhere.

Parents reported that their child felt disregarded when the HCPs did not listen to and take the child’s views seriously, but relied solely on what the blood tests displayed. The child had to clearly mark their feelings by, for example, crying and saying “*I can’t stand it. You have to do something now!”* before the HCP would take the child’s complaints seriously. One child said to the parent *“but they don’t understand me. Is this the way it is going to be?*. If the children reported problems and still did not receive help, they felt somewhat ignored and found the encounters pointless and did not repeat the information again.

*“You have to fill out a lot of surveys every time you go there, but they are never important. Or at least not something you feel is important to you, and you never hear anything back about them”* (Child Workshop 7).

Children found the encounters tiresome “*because when you don’t understand something, it’s pretty boring”* and perceived the discussions as non-important to the children themselves. When children did not understand health conversations, they could not keep up, did not know what to ask for, or how to formulate themselves: *“but then it’s pretty difficult to explain when they ask how things are”*. Children could solve poorly understood conversations by only repeating what the parents said, what the children had said in the past or hand over the communication to the parents instead.

“*Yes, my mom always comes with me to the hospital, and she kind of tells everything. Because they use such strange words, I don’t know what they mean”* (Child Workshop 1).

Both younger and older children had difficulty asking HCP about what they wanted to know and communicating their problems, at times. Parents perceived the children shy and children themselves said that they did not always dare to ask, and had difficulty in formulating and expressing their problems to the HCPs.

Parents were accustomed to observing their children and communicating with the doctor, but they were not informed of everything by the child, *“… yes, but he (the child) may have more pain than he says he has”*. Children protected their family and friends, by not telling exactly how bad they felt neither physically nor mentally since they did not want to upset or see them overreact. *“They get more worried and like … but what can we do? What can I do to help you? But there is nothing (they can do)”*. Children did not focus on daily pain because *“you don’t worry too much about it, because you know it will disappear. There is no need to, like… bring it up”*. Children and parents perceived that children needed to express their problems and how they felt to the parents, so they knew about these because *“it is quite the opposite that we (parents) do not really notice that he feels that bad, but he does”*.

Children also hid problems from HCPs and did not tell how bad they felt since they could be afraid of consequences, *“I usually say that I’m fine so I could avoid Methotrexate”*.

*“When I was little, I always said I was fine, it’s okay. It’s because you want to get away. So, when you are little, healthcare is not such a fun place. Nah. There are grown up people who do strange things with your body. So you do not really know what is happening”* (Child Workshop 5).

The children did not report current physical problems they knew would pass or troublesome occasions in the past, instead they focused on worsened problems which needed to be medicated. Mental problems were seen as separate from physical problems and *“I don’t think I really understood that it was something you could get help for”*, so children did not tell about such problems. Therefore, children found it desirable that HCPs asked them subtle questions about mental health, such as school and home conditions, sleeping and eating habits so HCPs could respond to such problems.

Sometimes children held problems within themselves for emotional reasons *“I think I wanted to be a little, sometimes a little tough too. Not so sentimental”*. It was easier for the children to explain when they showed problems instead of just talking about them during the encounters. Therefore, children found examinations by HCP valuable, in order to detect problems. Children did not always know which problems were related to JIA or not, and could believe that everyone else had similar problems.

*“I had problems in one of my elbows that I didn’t understand. I couldn’t stretch out completely, but I didn’t think about it, because it hadn’t been hurting me. Then I got a cortisone shot and that let me stretch the elbow again. I wouldn’t have found out about that if I hadn’t had a personal encounter*” (Child Workshop 8).

### Being at ease with healthcare encounters

This subtheme consists of the two sub-subthemes: “Being a normal event in life” and “Enabled by physical and mental space”. These describe how children felt more comfortable over time with coming to HCPs when they knew what would happen, and felt that HCPs gave them the necessary support they needed to participate.

The first healthcare encounter was particularly important and HCPs therefore needed to be extra nice and kind to the child, *“so the hospital is a safe zone”*. With more routine and older age the children felt safer *“because I am so used to it, so I know what is happening*”. Communicating problems to HCP was easier *“once you understand that they just want the best for me, then maybe you dare to open up a little bit more and say, ‘I’m actually hurting. I need help’”*. When the environment had a homely atmosphere and *“child-friendly”* furnishing, the children felt better about visiting the team. White walls, dull colors and dark rooms with *“such a creepy lighting”* was associated with *“those institutions in horror movies -will they eat me?”*.

Both children and parents had experienced that they had been well treated at healthcare encounters. When the child felt they were truly being paid attention to by the HCP who *“turned to him and then they said his name and asked about day-to-day things”* and *“they wave and say hello. They ask how it is, even if they are not my doctor for the day”*, the encounters felt personal and positive.

If HCP were dedicated and cheerful and “*dare to make a joke. Then it’s more fun to be there”.* Likewise, if the child was scared and got to meet the *“happiest person in the world. Then it immediately felt much, much better”*.

When HCP focused on the child’s interests and how they could cope with these, “*instead of just asking about pain and stiffness all the time”*, it became easier for the children to communicate their problems. Having confidence and trust in HCP was important. Children had more confidence in HCPs if they were calm, confident and positive, and communication and participation was facilitated if the families met the same HCP because *“it is difficult otherwise with the trust, that is where you can find each other”*. A new HCP for the child entailed that *“it can be a little difficult. And then I like hide behind my mom and let her talk”.* It was also easier for the children to open up and be honest if they felt that HCP understood them, that they believed in the children and did not blame them in any way. When children received positive results about their health, the encounters felt better.

*“At school, we have this kind of thing: if we are to give criticism, then you have to say two good things and one bad thing. I almost think it should be the same, like this: ‘yes this is great, but this is not so good’”* (Child Workshop 4).

Children could be more independent and participate at healthcare encounters if they knew what was going to happen and which and how many HCPs were going to attend. Children wanted to have the chance to decline students’ attendance in advance and wanted to know if they needed to undress during the encounter. Some children preferred to write down health concerns to remember at the encounters, while others did not need such preparations.

The parents perceived that preparations before their encounters, such as bringing pictures and videos showing the child’s problems, facilitated communications about the child’s problems. Likewise, an easy and short questionnaire that focused on the child’s interests, and going through these with HCP during the encounter could help. But the current health survey (Childhood health assessment questionnaire) used in clinical practice were perceived as not addressing the children needs and their problems and thus were perceived as “*completely insignificant to me”*.

Children wanted to tell themselves how they felt, because *“I don’t want my parents to tell completely. Because it is my body, it is in mine it hurts”*. The children needed time to think. *“I don’t know, it’s like I don’t understand. Or I just think for quite a while and then my mother answers instead. Because I haven’t figured out what to say”*. Over time, it became easier for children as they began to understand more, *“so it was like when I was eleven maybe. Then I began to grasp a little*”.

Children wanted and needed to discuss sensitive matters and behaviors, and some found it easier talk to the physician themselves without parents. When the child met HCPs without parents, the encounters felt different. The HCPs talked to the child in another way, and the child told HCPs more, asked questions, and learned other things.

*“But sometimes they can use more difficult words when the parents are there. While here, they’re trying to make it kind of personal. How do you say it, get to know me better?”* (Child Workshop 5).

However, it differed between which HCP children preferred to meet alone. If children did not need to formulate and tell things, they could meet HCPs by themselves more easily: *“If I’m just going to take medicine, then I can do it myself. And if I go to meet the doctor, then it can be good to have my parents with me”*. Children felt they remembered how they had felt, but they had to be more aware and more attentive during their own encounters without parents. Parents sometimes needed to accompany children to the encounters since the parents could remember other matters and had another overview of the child’s problems in the past. Therefore, the parents were able to remind the child and complement their information to HCPs.

“*But, when you are so young, then you have to help each other, because if you get the diagnosis when you are very little then you do not know what a normal condition is really. Then, it is good to receive help from your parents, since they also can help”* (Child Workshop 8).

At times children and parents described the children’s problems the same, and sometimes they needed to help each other to remember. Children found that their parents could exaggerate their problems at times, and they needed to correct the parents, *“it may be their description of how much pain I have sometimes, and how often. Sometimes I only have sore muscles from exercise while they think it is my joints”.*

So, *“in order to get the best care, it is important that both children and parents describe what they have seen and noticed” *(Child Workshop 8).

Children wanted to participate in both minor and major decisions, because *“I may feel that the medicine is also doing more, and that this medicine will help me if I can choose it and that may then help me feel mentally better because I’m active in choosing the medication”* (Child Workshop 8). However, the children wanted to leave some decisions to the HCP instead: *“but I feel like this, they really know best. Yes, so they must decide some”.*

Children had also felt they had been listened to in healthcare and *“I have always felt that my doctors have understood, have still tried to help me”*.

The HCPs efforts needed to be adapted to the situation, activities and interests of the child, in order to provide the child with tailored help. Exercises from a physiotherapist, for example, needed to have a clear goal and target what is important to the child and in a suitable way for the child. Otherwise, the child was not motivated to do the exercises, *“maybe the first day, but then I just, no I don’t have the energy”*. Encounters where the children were allowed to be physically active opened up for more communication because *“with the physiotherapist and occupational therapist, it feels like you can show more. And then it’s easier to explain”*.

Continuous information both verbally and in writing about the JIA diagnosis, the prognosis, examinations and treatments and why these were done were appreciated, as well as suggestions on where to find further information and were to turn to in case of problems.

*“I don’t always know so much about my disease and about me and how it affects me. Because I learned about it when I was so little that I have forgot… But I didn’t dare to ask as much when I was little”* (Child Workshop 6).

Likewise, thorough information about the medications, and what alternatives were available were appreciated. The participants wanted the information to be honest and with regard to the child’s maturity and development so the child could understand. Side-effects of medications were important for children to know since these could be hard to cope with.

*“It is also good, if you are new, that you can get information about your illness and about new medicines. That seems to me the most important thing about medication and side effects after I got Methotrexate. Does this make me nauseous or? If so, I did not intend to take it*” (Child Workshop 5).

It was also important that information was presented with a sense of hope and supplemented with what actions the child/parent could do to relieve problems. The HCP should ask; “have you (children and parents) understood?” after information sharing to ensure that everyone had understood.

## Discussion

Children may distance themselves from healthcare encounters if they find them emotionally stressful. Children felt more at ease with healthcare encounters, if they felt safe and strengthened, experienced personal and positive encounters and were given the appropriate space.

The results will be discussed using a model developed by Lundy to promote and structure children’s needs for the successful implementation of Article 12 in the UNCRC [23]. Although Lundy’s model was developed for educational settings, it is a useful model that outlines the four key concepts that should be considered for children’s participation in healthcare: Space, Voice, Audience and Influence [23].

The children wanted to actively participate in healthcare encounters, speak for themselves, participate in healthcare discussions and information exchange as much as possible. Other authors have concluded that children’s preferences for participation in healthcare situations can vary depending on their illness, competence, age, and type of decision to be made [15]. At times, children want to be active participants, and at times, they want to be passive bystanders. Parents and HCPs should therefore, view children as individuals with needs that vary according to each situation [15, 24]. Adolescents want to have a say in important health-related decisions and are usually not opposed to doctors asking personal questions in front of their parents [8, 25]. Some adolescents feel more independent and confident at healthcare encounters, while others feel less capable of self-management and want their parents present during encounters. Some adolescents lean on their parents because they feel uninvolved, lack confidence, and worry about their health, therefore, their parents’ presence support them in such situations [8].

The children in our study struggled with understanding medical language, discussions, and questions posed to them at healthcare encounters. Consequently, the children could not participate according to their wishes, and were reluctant to engage in healthcare encounters. The children did not have sufficient time to think about the questions they were asked and to formulate what they wanted to say. Adults, and especially HCPs involved at healthcare encounters, must, therefore, use their power wisely, and ensure the children are given the space they need and to understand the communication. Lundy describe that there should be an inclusive and safe space for the children without fear of rebuke [23]. The children said that as they became more experienced over time, and felt more familiar with healthcare, they could participate more, and were more involved in communications and decisions making.

These results relate to Lundy’s concept of *Space*. Children should be given opportunities to express their views, they should be asked about which matters they consider important, and how they would like to participate at a healthcare encounter [23]. HCPs should also address children directly and, avoid ‘talking over their heads’ [11]. The child has the right, not the duty, to express a view. It is important to remember that there might be occasions when children do not want to be involved, which should be respected too [7, 23].

The children found healthcare encounters emotionally stressful. They did not know what was going to happen, were afraid of hearing bad news, and they were exposed to situations they disliked. It is therefore important that HCPs are aware of these worries and stress factors among children. By giving children as much positive experience and support as possible, they may feel safer and more independent at healthcare encounters. Likewise, meeting the same HCP felt safer for the children, and enabled them to communicate more. In line with our results, Coyne has also found that children may avoid being active in healthcare encounters for various reasons; they fear bad news, do not want to cause trouble, do not want to disappoint HCPs, do not have sufficient time with HCPs, are unable to understand medical language, and are unable to understand their parents’ actions [7]. Gilljam et al. have also concluded that it is important to reduce children’s fears and uncertainty, to enhance children’s confidence, and increase their participation in healthcare encounters [12].

Children and parents responded that they wanted to be prepared and informed about plans for the healthcare encounters, so the children would feel safer. The children were more relaxed when they experienced personal and positive encounters, and when they felt safe at healthcare encounters. Children also have a right to privacy, and should be asked if additional HCPs can be present. A homely environment could also be calming to the children, which has been confirmed by Todres and Diaz [25]. Those authors also suggest that the physical space in child healthcare should be welcoming and appropriately furnished for all ages: colorful, well lit, clean and display messages that reflect diversity [25].

Two-way healthcare encounters between HCPs and adolescents without parents were experienced positive for children. Such encounters encouraged more child communication and relationship- building between the HCP and the child. Other authors have also found that adolescents with JIA, irrespective of their personality and confidence, preferred to talk to HCPs alone [25, 26]. Parents have a significant influence on whether children are involved at healthcare encounters or not [7]. Adolescents and their parents may differ in their preferences for child-physician interaction and the role of the parent during healthcare encounters [8]. Parents can have focuses and needs at encounters that do not match the focuses and needs of the child when it comes to communicating with HCPs [27]. Parents’ presence can inhibit adolescents from discussing sensitive topics or asking questions. Although adolescents appreciate their parents’ active involvement and support, they can find their interference annoying and redundant at three-way healthcare encounters [8]. Nevertheless, the children saw parents as complementary, since parents could refresh their child’s memory and complement the information they provided. However, at times the children perceived that their parents described their health worse than the children themselves experienced. Likewise in a qualitative study by author date, HCPs could perceive that children and parents have different ways of communicating the child’s health at healthcare encounters. Children were seen by HCPs to live in the moment, focusing on positive aspects, while the parents had a longer perspective and focused on the child’s problems [28]. The concept *Voice* relates to these findings. Children have the right to express their views freely, depending on their ability to form a view, whether mature or not [23]. In Lundy’s model the concept of ‘Voice’ relates to children’s rights to express their views freely, depending only on their ability to form their perspective, mature or not [23].

Both the children and parents requested more initial and continuous information about the disease, which symptoms were or were not related to JIA, the prognosis, and the medical treatment of JIA. It was not easy for the children to know which symptoms were related to JIA or not, and symptoms were normalised for children. In a thematic synthesis of qualitative studies [3], children with JIA wanted HCPs to deliver holistic care and ongoing current information about their illness, treatment, and procedures. Those children also wanted information about medical research and advances in JIA, to gain a sense of optimism for the future [3]. Also Van Dijkhuizen et al. have found that improvements were needed in the supportive care of patients with JIA and the accessibility of information [29]. The results of a Swedish Young Rheumatics report showed that members wanted more information on how their rheumatic disease and medical treatment affected them [30]. Even though another study showed limited evidence of increased knowledge among children leading to improved levels of child engagement during healthcare encounters [31], the children and parents in our study expressed a need for more information.

Both HCPs and parents must ensure that children can participate as much as they want in information sharing. In a study of children with cancer, most children wanted to be included and participate in information sharing. Nevertheless, at times, children want their parents to ‘filter’ the information to them instead, especially when the children feared hearing bad news, were reluctant to ask questions or felt ill [32]. Therefore, to keep children’s best interests at heart and to meet the children’s rights to information, HCPs should inform the child about the disease and its treatment, in an age- appropriate way and in accordance with the child’s wishes for information.

Parents and HCPs might withhold information to a child if they find the child vulnerable [32]. Another reason why families may lack desired information can be that HCPs are concerned about over-whelming families with information, especially at the beginning of disease onset [33]. Likewise, the children in our study responded that they could withhold information on how bad they really felt because of fear of the consequences of such disclosures. Nor did they want to worry or upset their family and friends. As a result, information may be restricted to everyone included at three-way healthcare encounters.

In relation to suitable methods for engaging children with long-term conditions in their healthcare, Bray et al. found that interventions have a role in improving the frequency and range of children’s engagement during encounters and can improve their satisfaction with their interactions, decisions and relationships with HCP´s during encounters [31]. An e-Health solution called Genia targeted at children with JIA seemed to improve the children’s awareness and self-management, helped empower children and to communicate personally relevant illness concerns with HCP [14]. However, the evidence relating to which intervention ‘works’ for children is limited. Bray et al. highlight the need for more participatory, engaging and child-centered interventions and methods [31].

Children had experienced not being taken seriously, listened to or understood by HCPs when describing problems. The child, consequently, felt disregarded and could not be offered the medical help needed. In contrast, other children had felt that the HCPs always listened to them and had perceived that HCPs could help them. Söderbäck et al. have pointed out that in order to capture a child’s perspective, parents and HCPs need to be attentive, sensitive, and supportive of each child’s expressions, experiences, and perceptions [34]. These results are related to the concept *Audience*, i.e. children’s rights to have their views actively listened to, not only heard, by those who make decisions. Effective observation and registering non-verbal cues children use at encounters when they express their views should be included [23].

The children in our study wanted to have a say in decisions related to their health, such as medications. Side-effects were important to know for the children, since these could be too difficult to cope with. In accordance with Tong et al. results [3], the participants in this study suggested that physiotherapy exercises needed to be enjoyable, integrated and related to their everyday living, their interests and leisure activities. Otherwise the children found the exercises boring, uninteresting and did not perform them.

Nevertheless, the children did not require full responsibility for decisions making, since they relied on HCPs’ knowledge. In the Swedish Young Rheumatics report, almost half of the respondents felt that healthcare did not work actively to make them feel joy of life or to be able to fulfil their dreams. Almost four out of ten were not satisfied with their medical treatment, and they experienced that they did not have any influence over the choices of medical treatment [30].

The concept of *Influence* relates to children’s views being ‘given due weight in accordance with their age and maturity’ [23]. Article 3 states that consideration of children’s best interest is required in decision-making. However, the formulation ‘given due weight’ in Article 12 is problematic since it is linked to ‘age and maturity’. Therefore, children’s communication and participation in healthcare encounters can be undermined by adults’, who may decide that children are not sufficiently mature to express their views [23, 35]. Children’s rights to have their views given due weight cannot be neglected on the basis that the adults in their lives know what is best for them [23].

### Methodological considerations

The discussions at the children’s workshops were short and condensed with much valuable information. Therefore, we found the qualitative content analysis (QCA) method suitable for this qualitative study [20]. QCA results are not considered as objective views about the world, but rather as the researchers’ interpretations of and interaction with the data and the results [19, 20].

When conducting QCA credibility, dependability and transferability are important measures for achieving trustworthiness [19, 20]. For enhanced credibility, the content of the workshops was decided upon between VL and AFW, and discussed with CE and TL. VL and AFW analysed the discussions at the workshops, and the results from the workshops were discussed in triangulation between researchers [16]. It would have been preferable with more participants. Several attempts with recruitment were made, but the recruitment of participants was challenging. Starting with individual interviews, followed by one or two workshops for children could have enabled the participation of more children. However, the included children did share valuable experiences with each other and the researchers. The young adults who participated in separate workshops added valuable information and shared their experiences of healthcare encounters as a child. According to Fadyl et al, participants will relate to both’ in-the-moment knowledge’ and ‘retrospective knowledge’, during interviews [36]. In our earlier study, HCPs found children and parents to complement each other, and that older children could recall their health better [28]. Children and adolescents in this study described that they could remember health events well, but their parents could explain these in more detail.

The results have been presented and discussed with participants for member check [16], and their suggestions and feedback on results were included in the analyses. The results were presented to research peers and colleagues in pediatrics and at an international conference for peer debriefing [16].

Another strength of the study was that the authors represented different professional perspectives including both ‘insider’ and ‘outsider perspectives’ [37]. VL, CE, TL, and IC have experience of working in paediatrics and working with children with JIA and were ‘insiders’ in the field. MS, RJ, and AFW were ‘outsiders’ in the field of paediatrics and working with children with JIA but they had experience of qualitative methods. However, we were all adults and, therefore, ‘outsiders’ in terms of children’s perspective.

For dependability, we had an open dialogue in the research team about the content of the workshops, and described the analysis process of the data thoroughly.

To facilitate transferability, we were transparent in the selection of informants and data collection with a thick description [16], and transparent about the analysis while still maintaining the anonymity of the participants. We also presented appropriate quotations. Our results are limited by the small sample size. However, this is an important and understudied area and this study provides valuable information for healthcare providers about children with JIA experiences of healthcare encounters.

## Conclusions

Children’s participation in healthcare encounters appeared to vary depending on if children feel alienated or familiar to the healthcare situations. Children distance themselves and are reluctant to healthcare encounters if they find them emotionally distressing, feel labelled as a ‘rheumatic patient’, and their views are disregarded during healthcare encounters. Children sometimes find it challenging to reveal how they really feel to both family and HCP for various reasons. In time children can become more familiar and at ease with healthcare situations when they feel safe and experience personal and positive encounters with HCPs. When children are prepared for what will happen, are provided with the space and support they need, and receive tailored help, they are more enabled to participate.

## Data Availability

The data including recordings and transcriptions have been stored at Umeå University, Physiotherapy and have not made publicly available due to the risk of identification of the participants.
